# Optimization of anesthetic decision-making in ERAS using Bayesian network

**DOI:** 10.3389/fmed.2022.1005901

**Published:** 2022-09-14

**Authors:** Yuwen Chen, Yiziting Zhu, Kunhua Zhong, Zhiyong Yang, Yujie Li, Xin Shu, Dandan Wang, Peng Deng, Xuehong Bai, Jianteng Gu, Kaizhi Lu, Ju Zhang, Lei Zhao, Tao Zhu, Ke Wei, Bin Yi

**Affiliations:** ^1^Chongqing Institute of Green and Intelligent Technology, Chinese Academy of Sciences (CAS), Chongqing, China; ^2^Department of Anesthesiology, The First Affiliated Hospital of Chongqing Medical University, Chongqing, China; ^3^Department of Anesthesiology, Southwest Hospital, Third Military Medical University, Chongqing, China; ^4^Department of Anesthesiology, Xuanwu Hospital, Capital Medical University, Beijing, China; ^5^Department of Anesthesiology, West China Hospital of Sichuan University, Chengdu, China

**Keywords:** Bayesian network, enhanced recovery after surgery, decision-making, gynecological tumor, machine learning

## Abstract

Enhanced recovery after surgery (ERAS) can accelerate patient recovery. However, little research has been done on optimizing the ERAS-related measures and how the measures interact with each other. The Bayesian network (BN) is a graphical model that describes the dependencies between variables and is also a model for uncertainty reasoning. In this study, we aimed to develop a method for optimizing anesthetic decisions in ERAS and then investigate the relationship between anesthetic decisions and outcomes. First, assuming that the indicators used were independent, the effects of combinations of single indicators were analyzed based on BN. Additionally, the impact indicators for outcomes were selected with statistical tests. Then, based on the previously selected indicators, the Bayesian network was constructed using the proposed structure learning method based on Strongly Connected Components (SCC) Local Structure determination by Hill Climbing Twice (LSHCT) and adjusted according to the expert’s knowledge. Finally, the relationship is analyzed. The proposed method is validated by the real clinical data of patients with benign gynecological tumors from 3 hospitals in China. Postoperative length of stay (LOS) and total cost (TC) were chosen as the outcomes. Experimental results show that the ERAS protocol has some pivotal indicators influencing LOS and TC. Identifying the relationship between these indicators can help anesthesiologists optimize the ERAS protocol and make individualized decisions.

## Introduction

Enhanced recovery after surgery (ERAS) is a clinical strategy designed to reduce patients’ stress responses to surgery and preserve their physiological state as much as possible. It includes a series of medical decisions in the perioperative period that can facilitate the rapid recovery of patients undergoing surgery. Implementing the ERAS protocol can result in shorter hospital stays, less postoperative pain, lower complication rates, and earlier initiation of feeding and ambulation (1, 2). For patients with different demographic characteristics, different treatment options, and postoperative complications, cooperation and specialization of multiple departments are required (3). ERAS has been applied in many departments (4–8), and previous ERAS studies have mainly verified its efficacy and safety of the ERAS protocol. Meanwhile, the feasibility and safety of monotherapy or equivalent measures have been investigated. A few studies explored the interaction between various indicators in ERAS and optimizing ERAS strategies.

Anesthetic decision-making is an integral part of the perioperative period and essential to ERAS implementation (9). The anesthesia decisions mainly involved intraoperative management and postoperative analgesia, such as intraoperative blood pressure, urine volume, non-steroidal drugs, and compound nerve blocks. There are significant links between patient recovery and anesthesia (10, 11). For gynecologic surgeries, the choice of anesthetic can influence postoperative outcomes. Prophylactic dexamethasone administration and propofol infusion have been shown to reduce the incidence of nausea and vomiting after laparoscopic gynecological operations (12, 13), as well as other studies related to pain control (14). However, no studies have systematically assessed the effects of a series of anesthesia decision indicators, the interaction between these indicators, or the overall influence of indicator combinations. In addition, some of the indicators may not be necessary. In clinical practice, optimizing the anesthetic part of ERAS and recognizing the interaction can enhance decision-making efficiency.

Optimizing the anesthetic decision-making in the ERAS protocol can be viewed as a multivariate analysis to determine the indicators that have a greater impact on the patient’s prognosis and recommend their combinations. For previous studies, traditional statistical methods mainly use multivariate analysis and logistic regression analysis to find indicators with statistical differences. However, the variable relationship in clinical decision-making is complex and uncertain, making it prone to problems such as insufficient fitting and low accuracy. Bayesian Network (BN) is a type of machine learning (ML) that has been applied to the medical field, such as disease diagnosis (15, 16), risk prediction (17, 18). Its core lies in probabilistic methods (19, 20). BN is a graphical model that describes the dependencies between data variables and is also a model for uncertainty reasoning. Based on probabilistic reasoning, BN can identify causal relationships between nodes and give the conditional probability of each node. Therefore, BN has advantages when variables have a relationship or uncertainty (21, 22). There is a lot of room for developing the BN algorithm for anesthesia decision-making in ERAS.

In this study, we aimed to develop a method for anesthesia decision optimization in ERAS and then investigate the relationship between anesthetic decisions and outcomes in patients with benign gynecological tumors based on the proposed method. By finding impact indicators in the anesthetic part of ERAS, we conducted a BN analysis of these indicators and made recommendations to improve the outcomes.

## Method description

**FIGURE 1:**
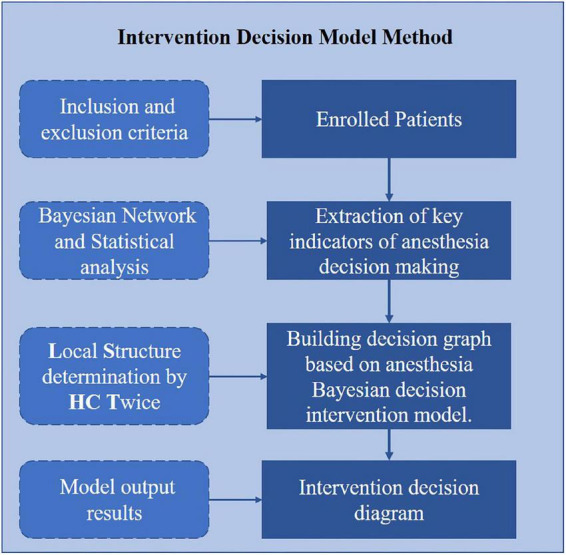
Study protocol.

Our method for anesthesia decision optimization in ERAS consists of two main steps: (1) extraction of key indicators of anesthesia decision making and (2) building a decision graph based on the anesthesia Bayesian decision intervention model. As shown in Figure 1, we first use Bayesian network and statistical tests to select indicators. Then, we propose a Bayesian decision intervention model for anesthesia based on the LSHCT method to construct a decision graph. Python 3.9 was used to construct the BN model.

The model is described in detail as follows:

### Extraction of key indicators of anesthesia decision making

Now, machine learning has been widely used in the field of computers, which can be optimized. They are applied to things such as Web service and feature extraction, including Quality-of-Service (QoS) prediction (23), improving the quality of online streaming feature selection (OSFS) with missing data (24), handling the data streams with a varying feature space based on Generative Learning with Streaming Capricious (GLSC) (25), etc. ML can also be used in the optimization of medical decision-making. In this study, we chose the Bayesian Network (BN). BN is a graphical model to describe the dependency relationship between data variables, as well as a model for uncertainty reasoning. BN provides a convenient framework for people to express the dependency relationship, making the logic of uncertainty reasoning clearer and more understandable. From a statistical point of view, BN is a graphical probability model. Pearl et al. from the University of California proposed the Bayesian Network model for the first time in 1988 (26). They applied BN to the expert system successfully, making it a popular method for uncertain expert knowledge and reasoning.

BN consists of two parts: network structure and network parameters. The network structure of BN is a Directed Acyclic Graph (DAG). Each node in the DAG represents a variable in a specific domain. The directed arc between nodes represents the dependency relationship among nodes, which is the qualitative characterization of the relationship between nodes. Each node has a conditional probability distribution function associated with it (the node without a parent node is represented as a prior distribution function), namely the network parameter, which quantitatively describes the dependency relationship between the variable node and its parent node. If the variable is discrete, the network parameters are represented as conditional probability tables of this node for a given parent node. In BN, there is no specific input node or output node, and any node can be used as an input node or output node.

Definition: a directed acyclic graph that meets the following four conditions is called the Bayesian Network (27):

(1) There is a set of variables *V* = {*X*_*i*_}, *i* = 1,2,⋯,*n*, and there is the set of directed edges between the corresponding nodes of variables *E*;
(2) The value of each variable can be either discrete or continuous;
(3) A directed acyclic graph *G* = < *V*,*E* > is formed by the nodes corresponding to variables and the directed edges between nodes, where *V* is the node-set, and *E* is the directed edge set, reflecting the causal dependence between nodes;
(4) A conditional probability distribution table *P*(*X*_*i*_|*Pa*(*X*_*i*_)) is corresponding to each node *X_i* and its parent node set *Pa*(*X*_*i*_), and it satisfies $P\,\left(X_{1},X_{2},\cdots ,X_{n}\right)=\prod_{i=1}^{n}P\,\left(X_{i}|P\,a\,\left(X_{i}\right)\right)$.

The BN has been widely used in clinical diagnosis (28) and gene analysis (29). The main research content of the BN includes structure learning, parameter learning, and inference. Among them, structure learning is the basis of parameter learning and inference. BN structure learning refers to using the data sample set and combining the prior knowledge as much as possible to obtain the optimal topological structure. Learning BNs from data is a special case of ML. The approximate learning methods of learning BN structure from complete data include the conditional independence test-based method, the score search-based method, and the hybrid method (30). The method based on score search regards network structure learning as an optimization problem, and its goal is to search for the network structure with the highest score. With the increasing of the nodes number, the network structure space to be searched increases exponentially, which has been proved to be an NP-Hard problem (31). Heuristic search is usually used, which makes it easy to fall into an optimal local solution. This study proposed a new structural learning method based on the idea of divide and conquer.

**FIGURE 2:**
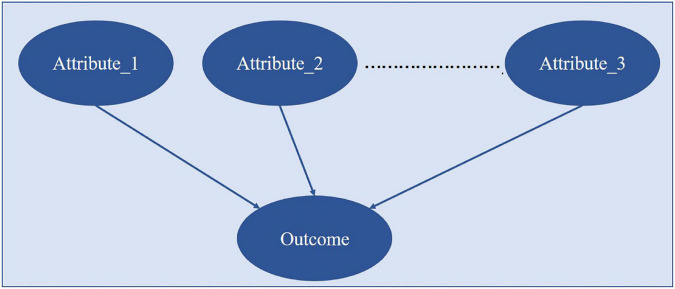
The analysis of indicator combination of BN structure.

The data of the enrolled patients were used to construct a simple BN, which assumed that the indicators used were independent of each other, as shown in Figure 2. The BN parameters were learned by the Bayesian estimation method with the clinical case dataset. The effects of combinations of single indicators on outcomes were analyzed, and the method gave the groups’ results and their probabilities. The probability of state-0 (optimal state) of the outcome node in the conditional probability table was ranked, and the combinations of values of the corresponding indicators with a probability greater than 0.9 were retained. Then, the influence of single indicator nodes would be calculated. To select the indicators and their grades:

(1) Sort each indicator by *p* values;
(2) Choose the grade of each indicator that accounted for the largest proportion of postoperative LOS-state-0 and TC-state-0, where LOS, where LOS and TC are the abbreviations of “*length of stay”* and “*total cost,”* respectively, and state-0 is the optimal state of corresponding indicators;
(3) Combined the indicators with specific grades starting from the one with the smallest *P* value until the last one;
(4) Perform a screening in the retained groups (probability > 0.9);
(5) Perform a statistical test and combine the selected indicators with statistical differences.

### Anesthesia Bayesian decision intervention model based on the LSHCT method

With the results of indicator selection, we combined an improved BN structure learning method based on Strongly Connected Components (SCC) Local Structure determination by Hill Climbing Twice (LSHCT) and expert knowledge to build a BN and optimized anesthesia decision-making in ERAS. This step used BN to analyze the relationship between the selected indicators. First, the BN structure is automatically learned from clinical data by LSHCT and then adjusted reasonably based on experts’ experience.

**FIGURE 3:**
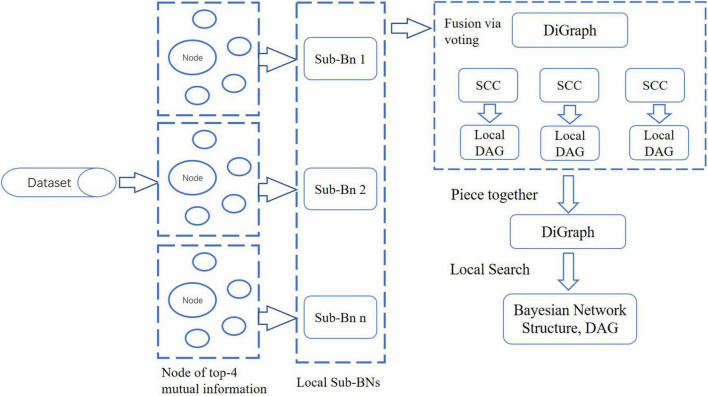
Flow diagram of the LSHCT method.

In BN, each node represents a random variable or data indicator. In this paper, with a divide-and-conquer strategy, we proposed a novel BN structure learning method by LSHCT. This is a score-based search method, and its core idea is to split the overall construction of the BN structure into the sub-BNs construction, fusion, and revision, as shown in Figure 3.

LSHCT consists of the following steps:

(1) Calculate the mutual information matrix between each pair of nodes, which is a symmetric matrix.
(2) For each node, select the four nodes with the most mutual information, and build a sub-BN for these five nodes.
(3) Piece together all the sub-BNs. The direction of the edge between a pair of nodes is determined by voting when both directions exist. And then, we obtained a directed graph which may not be a directed acyclic graph (DAG).
(4) Search all SCC in the directed graph obtained in the previous step.
(5) Convert each SCC into an undirected graph as the skeleton of the local structure. Determine the direction of the edges in the skeleton by the Hill Climbing (HC) algorithm twice. For the first time, the skeleton is used as the white list of the HC algorithm by converting each edge of the skeleton into two edges in opposite directions. For the second time, the result of our previous HC algorithm is used as the initial network structure.
(6) After determining the local structure of all SCC, it is already a DAG as a whole. Taking this as the initial network structure, we further use the local search method to obtain the network structure with a maximum score as the final result.

The Bayesian Network constructed by the LSHCT method was adopted to analyze the association relationship between selected indicators. The process consists of the following steps:

Step 1: According to the above methods, select indicator sets based on *p* values for different outcomes.
Step 2: The corresponding data sets were constructed by integrating indicators and outcomes according to different outcomes (postoperative hospitalization duration/total cost).
Step 3: Data preprocessing (same as the first use of the BN). As all the indexes were nominal, there was no need to preprocess the data for each indicator. The outcomes are of numerical type, which we assigned to two categories based on the mean value. One feature of Bayesian networks is that they allow the presence of missing values, so we retain the missing values in the dataset.
Step 4: Learn the BN structure by the LSHCT method, and output the graph of the BN structure, namely the dependency relationship between selected indicators.

## Experiment and results

### Dataset

This study conducted an experiment in three hospitals in China (Southwest Hospital of Third Military Medical University, Xuan Wu Hospital of Capital Medical University, and West China Hospital of Sichuan University). According to the regulation of the People’s Republic of China on the administration of human genetic resources, the collection of raw data was subject to approval by the science and technology administrative department of the state council (AQ: 2020SQCJ7444). Ethical approval was obtained, and the informed consent requirement was waived (Certification Number: KY201936, certification number: 2019-132, and certification number: 2021-349, respectively). The registration has been completed on the website of the China Clinical Trial Registry Center (ChiCTR1900023927).

### Inclusion and exclusion criteria

Inclusion criteria were as follows: (1) age: 18–80 years old; (2) Patients who underwent laparoscopic myomectomy and bilateral uterine adnexectomy and were diagnosed as benign gynecological tumors; (3) general anesthesia (without compound another anesthesia method). Moreover, the exclusion criteria were as follows: (1) patients with severe heart, lung, and kidney diseases before the operation, ASA grade ≥ 4; (2) patients with systemic metastases of malignant tumors; patients undergoing extensive hysterectomy + omentum resection + pelvic lymph node dissection; (3) patients who were converted from laparoscopic to laparotomy during surgery; patients undergoing the surgery which involved greater than or equal to two surgical departments; (4) patients undergoing rescue measures for critical diseases such as cardiac arrest, liver failure, and anaphylactic shock during the operation; and (5) patients with a large amount of incomplete medical records.

### Patients and data collection

**FIGURE 4:**
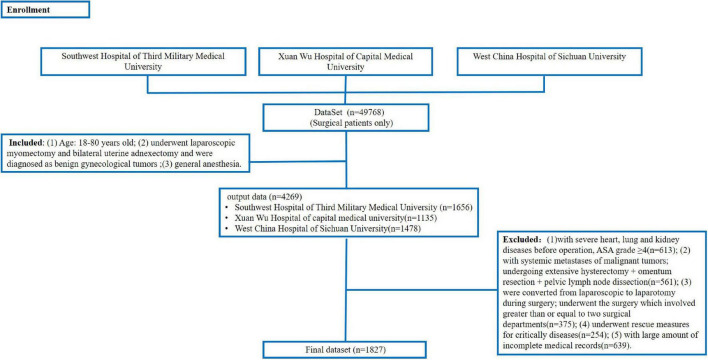
The process of enrollment.

A dataset (*n* = 49,768, surgical patients only) constructed from three hospitals was used to search for patients according to inclusion and exclusion criteria. Finally, we enrolled 1,827 patients. The detailed process of data collection is shown in Figure 4. The figure shows the protocol for constructing the dataset (*n* = 49,768) and outputting the patients (*n* = 4,269) with the inclusion criteria. Then, we excluded the patients (*n* = 2,442) according to the exclusion criteria. Finally, we got 1,827 patients.

The information about these patients included two parts: (1) general information and perioperative data of patients: patient’s ID number, medical record number, age, height, weight, and other information such as preoperative hemoglobin (Hb), preoperative systolic blood pressure (SBP), preoperative diastolic blood pressure (DBP), preoperative mean blood pressure (MBP), length of operation, intraoperative urine volume, intraoperative colloid volume, intraoperative crystalloid volume, intraoperative blood gas times, postoperative Hb; (2) indicators related to anesthesia. We initially sorted out more than 50 questions related to anesthetic decision-making, according to previous domestic and foreign literature reports combined with the clinical practice. Through the screening process by panels of anesthesiologists and gynecologists, we finally determined 18 indicators that obtained attention with more than 50%. The outcomes were decided as “postoperative length of stay (LOS)” and “total cost (TC).” The meaning and explanation of these indicators and outcomes are described in Table 1.

**TABLE 1:** The meaning and explanation of the value of each indicator.

Indicator	Grading	Meaning and explanation
Urine volume _ length of operation _100	Low	Urine volume/length of operation < 100
	High	≥ 100
Whether to use dexamethasone	N	No
	Y	Yes
Intraoperative use of sevoflurane	N	No
	Y	Yes
Intraoperative use of propofol	N	No
	Y	Yes
Crystalloid solution: colloid solution ratio −2:1	Low	No colloid solution use
	High	Crystalloid solution: colloid solution ratio > 2:1
	Less	Crystalloid solution: colloid solution ratio < 2:1
Whether to use blood transfusion in 6–9 g hemoglobin	0	Whether to use blood transfusion when in [60, 90], no, in the range
	1	Whether to use blood transfusion when in [60, 90], yes, in the range
	2	Whether to use blood transfusion when in [60, 90], no, not, in the range
Whether to use the dexmedetomidine	N	No
	Y	Yes
Intraoperative systolic blood pressure (SBP)	Low	Below 20%, compared to preoperative period
	Normal	−20%∼+20%
	High	Above 20%
Intraoperative diastolic blood pressure (DBP)	Low	Below 20%, compared to preoperative period
	Normal	−20%∼+20%
	High	Above 20%
Intraoperative mean blood pressure (MBP)	Low	Below 20%, compared to the preoperative period
	Normal	−20%∼+20%
	High	Above 20%
Intraoperative ETCO_2_	0	<35
	1	35–45
	2	45–55
	3	55–60
Positive balance	0	0–500
	1	500–1,000
	2	1,000–1,500
	3	1,500+
Intraoperative use of myocardial nutritional drugs	N	No
	Y	Yes
Intraoperative use of hemostatic	N	No
	Y	Yes
Postoperative compound nerve block analgesia	N	No
	Y	Yes
Whether to use opioids as dominance in postoperative analgesia (fentanyl)	N	No
	Y	Yes
Whether to use non-steroidal drugs in postoperative analgesia	N	No
	Y	Yes
Whether to use a muscle relaxant antagonist	N	No
	Y	Yes
Postoperative length of stay, LOS	Less	<4.546
	More	≥ 4.546
Total cost, TC	Less	<17,548.3355
	More	≥ 17,548.3355

### Statistical analysis of key indicators of anesthesia decision-making

Measurement data were recorded as mean ± standard deviation or median (interquartile range). A *T*-test or Mann–Whitney *U*-test was used to analyze the differences between measurement data. The chi-square or Fisher’s exact test were used to analyze the differences between counting data. All the tests were two-sided, and *P* < 0.05 was considered statistically significant. SPSS25.0 statistical software was used for analysis.

Our study ultimately analyzed 1,827 patients and grouped them according to the outcomes: LOS-state 0 < 4.546 d, state 1 ≥ 4.546 d; TC-state-0 < 17,548.3355 Yuan, and state-1 ≥ 17,548.3355 Yuan. The probability that the LOS is less than 4.546 days was 62.56% (1,143/1,827), and the probability that the TC is less than 17,548.3355 Yuan was 54.02% (987/1,827).

For the general condition of the patients, most characteristics of the patients were statistically significant, except for the preoperative SBP, preoperative DBP, and preoperative MBP between LOS groups and the BMI, preoperative Hb, and preoperative DBP between TC groups, *P* > 0.05, as shown in Table 2.

**TABLE 2:** The comparisons of the general conditions of patients and the indicators associated with anesthetic decision-making.

Indicator*		All	LOS < 4.546 d	LOS ≥ 4.546 d	P	TC < 17,548.33 55	TC ≥ 17,548.33 55	P
Age (year)		47 (7)	47 (6)	46 (8)	0.018	46 (7)	48 (7)	0.000^†^
BMI (kg/m^2^)		23.4 (4.2)	23.6 (4.4)	23.2 (3.8)	0.000^†^	23.4 (4.4)	23.6 (3.8)	0.465
Preoperative Hb (g/L)		112 (30)	113 (27)	110 (33)	0.006	112 (23)	111.5 (36)	0.106
Preoperative SBP (mmHg)		124 (22)	124 (24)	123 (19)	0.332	125 (23)	122 (20)	0.000^†^
Preoperative DBP (mmHg)		76 (17)	76 (16)	75 (14)	0.093	75.5 (16)	76 (16)	0.752
Preoperative MBP (mmHg)		91.3 (17.0)	91.3 (17.3)	91 (15)	0.148	91.7 (17.0)	91.2 (16.8)	0.043
Length of operation (min)		110.0 (66.8)	105.0 (55.0)	129 (71)	0.000^†^	108.0 (55.5)	115.0 (80.0)	0.000
Intraoperative urine volume (ml)		200 (100)	200 (100)	200 (100)	0.001	200 (100)	200 (100)	0.002
Intraoperative colloid volume (ml)		350 (500)	300 (500)	500 (500)	0.000^†^	300 (500)	500 (500)	0.000
Intraoperative crystalloid volume (ml)		750 (400)	750 (350)	750 (400)	0.008	750 (400)	750 (400)	0.001
Intraoperative blood gas times (times)		0 (1)	0 (1)	0 (1)	0.004	0 (1)	0 (1)	0.000
Postoperative Hb (g / L)		104 (26)	105 (25)	100 (28)	0.000^†^	106 (25)	99 (27)	0.000
LOS (day)		4 (1)	4 (1)	5 (1)	0.000^†^	4 (2)	4 (2)	0.000^†^
TC (Yuan)		17,309.67 (4,166.82)	16,485.27 (3,876.02)	18,363.42 (4,612.88)	0.000^†^	15,440.2 (2,360.0)	19,654.65 (2,775.56)	0.000^†^
Urine volume_ length of operation_100	Urine/length of operation < 100	600	333 (29.1%)	267 (39.4%)	0.000^†^	321 (32.5%)	282 (33.7%)	0.597
	≥100	1,221	810 (70.9%)	411 (60.6%)		666 (67.5%)	555 (66.3%)	
Whether to use dexamethasone	No	1,380	852 (74.5%)	528 (77.5%)	0.150	663 (67.2%)	717 (85.4%)	0.000^†^
	Yes	444	291 (25.5%)	153 (22.5%)		324 (32.8%)	123 (14.6%)	
Intraoperative use of sevoflurane	No	1,482	921 (80.6%)	561 (82.4%)	0.340	801 (81.2%)	684 (81.4%)	0.881
	Yes	342	222 (19.4%)	120 (17.6%)		186 (18.8%)	156 (18.6%)	
Intraoperative use of propofol	No	144	99 (8.7%)	45 (6.6%)	0.116	78 (7.9%)	66 (7.9%)	0.971
	Yes	1,680	1,044 (91.3%)	636 (93.4%)		909 (92.1%)	774 (92.1%)	
Crystalloid solution: colloid solution ratio −2:1	No colloid solution use	651	444 (38.8%)_‡_	207 (30.4%)_‡_	0.000^†^	429 (43.5%)_‡_	225 (26.8%)_‡_	0.000^†^
	> 2:1	330	186 (16.3%)_§_	144 (21.1%)_§_		138 (14%)_§_	192 (28.2%)_§_	
	< 2:1	843	513 (44.9%)_§_	330 (48.5%)_§_		420 (42.6%)_| |_	423 (50.4%)_| |_	
Whether to use blood transfusion in 6–9 g hemoglobin	no, in [60, 90]	114	69 (6%)	45 (6.6%)	0.183	15 (1.5%)_a_	99 (11.8%)_a_	0.000^†^
	yes, in [60, 90]	36	18 (1.6%)	18 (2.6%)		0 (0%)_a_	36 (4.3%)_a_	
	no, not in [60, 90]	1,713	1,095 (92.4%)	618 (90.7%)		972 (98.5%)_§_	705 (83.9%)_§_	
Whether to use the dexmedetomidine	No	363	249 (21.8%)	114 (16.7%)	0.009	309 (31.3%)	54 (6.4%)	0.000^†^
	Yes	1,461	894 (78.2%)	567 (83.3%)		678 (68.7%)	786 (93.6%)	
Intraoperative systolic blood pressure	Below 20%	327	183 (16.1%)_‡_	144 (21.2%)_‡_	0.017	207 (21%)_‡_	123 (14.7%)_‡_	0.000
	−20%∼+20%	1,434	918 (80.5%)_§_	516 (76.1%)_§_		765 (77.7%)_§_	669 (79.9%)_§_	
	Above 20%	57	39 (3.4%)_‡_,_§_	18 (2.7%)_‡_,_§_		12 (1.2%)_| |_	45 (5.4%)_| |_	
Intraoperative diastolic blood pressure	Below 20%	303	171 (15%)_‡_	132 (19.4%)_‡_	0.005	168 (17%)_‡_	135 (16.1%)_‡_	0.000^†^
	−20%∼+20%	1,431	924 (80.8%)_§_	507 (74.4%)_§_		789 (79.9%)_‡_	645 (76.8%)_‡_	
	Above 20%	90	48 (4.2%)_‡_,_§_	42 (6.2%)_‡_,_§_		30 (3%)_§_	60 (7.1%)_§_	
Intraoperative mean blood pressure	Below 20%	201	105 (9.2%)_‡_	96 (14.2%)_‡_	0.001	126 (12.8%)_‡_	75 (9%)_‡_	0.000^†^
	−20%∼+20%	1,536	990 (86.8%)_§_	546 (80.5%)_§_		837 (85.1%)_‡_	702 (83.9%)_‡_	
	Above 20%	81	45 (3.9%)_‡_,_§_	36 (5.3%)_‡_,_§_		21 (2.1%)_§_	60 (7.2%)_§_	
Intraoperative ETCO_2_	< 35	1,503	693 (60.6%)_‡_	360 (52.9%)_‡_	0.000^†^	627 (63.5%)_‡_	429 (51.1%)_‡_	0.000^†^
	35–45	768	450 (39.4%)_§_	318 (46.7%)_§_		360 (36.5%)_§_	408 (48.6%)_§_	
	45–55	3	0 (0%)_§_	3 (0.4%)_§_		0 (0%)_‡_,_§_	3 (0.4%)_‡_,_§_	
Positive balance	0–500	318	231 (20.2%)_‡_	87 (12.8%)_‡_	0.000^†^	195 (19.8%)_‡_	123 (14.6%)_‡_	0.001^†^
	500–1,000	1,197	729 (63.8%)_§_	468 (68.7%)_§_		636 (64.4%)_§_	564 (67.1%)_§_	
	1,000–1,500	276	168 (14.7%)_§_	108 (15.9%)_§_		147 (14.9%)_‡_,_§_	129 (15.4%)_‡_,_§_	
	1,500+	33	15 (1.3%)_§_	18 (2.6%)_§_		9 (0.9%)_| |_	24 (2.9%)_| |_	
Intraoperative use of myocardial nutritional drugs	No	1,641	1,038 (90.8%)	603 (88.5%)	0.119	960 (97.3%)	684 (81.4%)	0.000^†^
	Yes	183	105 (9.2%)	78 (11.5%)		27 (2.7%)	156 (18.6%)	
Intraoperative use of hemostatic	No	1,185	735 (64.3%)	450 (66.1%)	0.442	597 (60.5%)	588 (70%)	0.000^†^
	Yes	639	408 (35.7%)	231 (33.9%)		390 (39.5%)	252 (30%)	
Postoperative compound nerve block analgesia	No	1,053	687 (60.1%)	366 (53.7%)	0.008	504 (51.1%)	549 (65.4%)	0.000^†^
	Yes	771	456 (39.9%)	315 (46.3%)		483 (48.9%)	291 (34.6%)	
Whether to use opioids as dominance in postoperative analgesia (fentanyl)	No	1,767	1,119 (97.9%)	648 (95.2%)	0.001	966 (97.9%)	804 (95.7%)	0.008
	Yes	57	24 (2.1%)	33 (4.8%)		21 (2.1%)	36 (4.3%)	
Whether to use non-steroidal drugs in postoperative analgesia	No	1,023	618 (54.1%)	405 (59.5%)	0.025	369 (37.4%)	654 (77.9%)	0.000^†^
	Yes	801	525 (45.9%)	276 (40.5%)		618 (62.6%)	186 (22.1%)	
Whether to use muscle relaxant antagonist	No	435	273 (23.9%)	162 (23.8%)	0.963	264 (26.7%)	171 (20.4%)	0.001
	Yes	1,389	870 (76.1%)	519 (76.2%)		723 (73.3%)	669 (79.6%)	

*Non-normally distributed measurement data were expressed as Median (interquartile range).

^†^*P* = 0.000 refers to *P* < 0.001.

_‡, §, ||_ Each subscript letter represents a subset of the indicator class, with no significant difference between the horizontal columns at the 0.05 level.

LOS, length of stay; TC, total cost; BMI, body mass index; Hb, hemoglobin; SBP, systolic blood pressure; DBP, diastolic blood pressure; MBP, mean blood pressure; ETCO2, end tidal carbon dioxide.

Among the 18 indicators associated with anesthesia decision-making, 11 indicators, including “urine volume_ length of operation_100,” “crystalloid solution: colloid solution ratio −2:1,” “whether to use dexmedetomidine,” “intraoperative SBP,” “intraoperative DBP,” “intraoperative MBP,” “intraoperative ETCO2,” “positive balance,” “postoperative compound nerve block analgesia,” “whether to use opioids as dominance in postoperative analgesia (fentanyl)” and “whether to use non-steroidal drugs in postoperative analgesia,” had significant differences between LOS groups, *P* < 0.05, as shown in Table 2. There were 15 indicators in TC groups that made a statistical difference, *P* < 0.05, as shown in Table 2.

### Selection results of key indicators for anesthesia decision-making

There were 18 indicators in total. The BN shown in Figure 2 was used to learn model parameters based on the case dataset and then rank the indicator combinations by the state-0 (optimal state) probability of the resulting node according to the conditional probability table. We retained the combinations with a probability value > 0.9. There were 957 combinations in LOS-state-0 and 558 combinations in LOS-state 1. There were 840 combinations in state-0 and 681 groups in state-1 (sub-optimal state) for the TC group. We conducted a single indicator analysis of these groups, as shown in Tables 3, 4.

**TABLE 3:** The influence of Bayesian indicator nodes for the postoperative length of stay.

Indicator node		Postoperative length of stay−0*−(957 cases)	Postoperative length of stay −1*−(558 cases)	*P*
Whether to use blood transfusion in 6–9 g hemoglobin	0	69 (7.21%)	45 (8.06%)	0.255389
	1	15 (1.57%)	15 (2.69%)	
	2	873 (91.22%)	498 (89.25%)	
Urine volume _ length of operation _100	Low	315 (32.92%)	252 (45.16%)	0.000002
	High	642 (67.08%)	306 (54.84%)	
Whether to use the dexmedetomidine	N	216 (22.57%)	105 (18.82%)	0.084633
	Y	741 (77.43%)	453 (81.18%)	
Whether to use dexamethasone	N	696 (72.73%)	417 (74.73%)	0.394152
	Y	261 (27.27%)	141 (25.27%)	
Whether to use a muscle relaxant antagonist	N	249 (26.02%)	150 (26.88%)	0.713013
	Y	708 (73.98%)	408 (73.12%)	
Crystalloid solution: colloid solution ratio −2:1	Low	381 (39.81%)_‡_	168 (30.11%)_‡_	0.000289
	High	171 (17.87%)_§_	132 (23.66%)_§_	
	Less	405 (42.32%)_§_	258 (46.24%)_§_	
Intraoperative ETCO_2_	0	591 (61.76%)_‡_	294 (52.69%)_‡_	0.000157
	1	366 (38.24%)_§_	261 (46.77%)_§_	
	2	0 (0%)_§_	3 (0.54%)_§_	
Intraoperative use of sevoflurane	N	750 (78.37%)	444 (79.57%)	0.581417
	Y	207 (21.63%)	114 (20.43%)	
Intraoperative use of propofol	N	90 (9.4%)	39 (6.99%)	0.104249
	Y	867 (90.6%)	519 (93.01%)	
Intraoperative mean blood pressure	Low	96 (10.03%)_‡_	93 (16.67%)_‡_	0.000322
	Normal	816 (85.27%)_§_	432 (77.42%)_§_	
	High	45 (4.7%)_‡_,_§_	33 (5.91%)_‡_,_§_	
Intraoperative systolic blood pressure	Low	171 (17.87%)_‡_	141 (25.27%)_‡_	0.002418
	Normal	747 (78.06%)_§_	399 (71.51%)_§_	
	High	39 (4.08%)_‡_,_§_	18 (3.23%)_‡_,_§_	
Intraoperative use of myocardial nutritional drugs	N	852 (89.03%)	480 (86.02%)	0.083242
	Y	105 (10.97%)	78 (13.98%)	
Intraoperative use of hemostatic	N	597 (62.38%)	351 (62.9%)	0.839885
	Y	360 (37.62%)	207 (37.1%)	
Intraoperative diastolic blood pressure	Low	156 (16.3%)_‡_	123 (22.04%)_‡_	0.001814
	Normal	756 (79%)_§_	396 (70.97%)_§_	
	High	45 (4.7%)_‡_,_§_	39 (6.99%)_‡_,_§_	
Postoperative compound nerve block analgesia	N	558 (58.31%)	285 (51.08%)	0.006276
	Y	399 (41.69%)	273 (48.92%)	
Whether to use opioids as dominance in postoperative analgesia (fentanyl)	N	933 (97.49%)	525 (94.09%)	0.000778
	Y	24 (2.51%)	33 (5.91%)	
Whether to use non-steroidal drugs in postoperative analgesia	N	483 (50.47%)	309 (55.38%)	0.065167
	Y	474 (49.53%)	249 (44.62%)	
Positive balance	0	216 (22.57%)_‡_	81 (14.52%)_‡_	0.000437
	1	570 (59.56%)_§_	363 (65.05%)_§_	
	2	156 (16.3%)_§_	96 (17.2%)_§_	
	3	15 (1.57%)_§_	18 (3.23%)_§_	

_‡_, _§_ Each subscript letter represents a subset of the indicator class, with no significant difference between the horizontal columns at the 0.05 level.

*Postoperative length of stay-state-0 (<4.546 d). Postoperative length of stay-state-1 (≥ 4.546 d).

**TABLE 4:** The influence of Bayesian indicator nodes on the total cost.

Indicator node		Total cost−0*−(840 cases)	Total cost−1*−(681 cases)	*P*
Whether to use blood transfusion in 6–9 g hemoglobin	0	15 (1.79%)_‡_	99 (14.54%)_‡_	4.4001E-30
	1	0 (0%)_‡_	33 (4.85%)_‡_	
	2	825 (98.21%)_§_	549 (80.62%)_§_	
Urine volume _ length of operation _100	Low	312 (37.14%)	267 (39.21%)	0.409685
	High	528 (62.86%)	414 (60.79%)	
Whether to use the dexmedetomidine	N	273 (32.5%)	51 (7.49%)	2.2441E-32
	Y	567 (67.5%)	630 (92.51%)	
Whether to use dexamethasone	N	549 (65.36%)	567 (83.26%)	4.0022E-15
	Y	291 (34.64%)	114 (16.74%)	
Whether to use a muscle relaxant antagonist	N	243 (28.93%)	144 (21.15%)	0.000529
	Y	597 (71.07%)	537 (78.85%)	
Crystalloid solution: colloid solution ratio −2:1	Low	363 (43.21%)_‡_	174 (25.55%)_‡_	3.2005E-13
	High	129 (15.36%)_§_	174 (25.55%)_§_	
	Less	348 (41.43%)_*c*_	333 (48.9%)_*c*_	
Intraoperative ETCO_2_	0	549 (65.36%)_‡_	348 (51.1%)_‡_	1.2488E-8
	1	291 (34.64%)_§_	330 (48.46%)_§_	
	2	0 (0%)_‡_,_§_	3 (0.44%)_‡_,_§_	
Intraoperative use of sevoflurane	N	669 (79.64%)	534 (78.41%)	0.557875
	Y	171 (20.36%)	147 (21.59%)	
Intraoperative use of propofol	N	69 (8.21%)	57 (8.37%)	0.912737
	Y	771 (91.79%)	624 (91.63%)	
Intraoperative mean blood pressure	Low	117 (13.93%)_‡_	66 (9.69%)_‡_	1.8525E-7
	Normal	702 (83.57%)_‡_	558 (81.94%)_‡_	
	High	21 (2.5%)_§_	57 (8.37%)_§_	
Intraoperative systolic blood pressure	Low	195 (23.21%)_‡_	114 (16.74%)_‡_	2.8249E-8
	Normal	633 (75.36%)_§_	522 (76.65%)_§_	
	High	12 (1.43%)_*c*_	45 (6.61%)_*c*_	
Intraoperative use of myocardial nutritional drugs	N	813 (96.79%)	525 (77.09%)	8.0189E-32
	Y	27 (3.21%)	156 (22.91%)	
Intraoperative use of hemostatic	N	486 (57.86%)	462 (67.84%)	0.000064
	Y	354 (42.14%)	219 (32.16%)	
Intraoperative diastolic blood pressure	Low	156 (18.57%)_‡_	117 (17.18%)_‡_	0.000068
	Normal	657 (78.21%)_‡_	507 (74.45%)_‡_	
	High	27 (3.21%)_§_	57 (8.37%)_§_	
Postoperative compound nerve block analgesia	N	408 (48.57%)	429 (63%)	1.8753E-8
	Y	432 (51.43%)	252 (37%)	
Whether to use opioids as dominance in postoperative analgesia (fentanyl)	N	819 (97.5%)	645 (94.71%)	0.004439
	Y	21 (2.5%)	36 (5.29%)	
Whether to use non-steroidal drugs in postoperative analgesia	N	279 (33.21%)	510 (74.89%)	7.4736E-59
	Y	561 (66.79%)	171 (25.11%)	
Positive balance	0	189 (22.5%)_‡_	111 (16.3%)_‡_	0.000324
	1	501 (59.64%)_§_	432 (63.44%)_§_	
	2	141 (16.79%)_‡_,_§_	114 (16.74%)_‡_,_§_	
	3	9 (1.07%)_*c*_	24 (3.52%)_*c*_	

_‡_, _§_, _c_ Each subscript letter represents a subset of the indicator class, with no significant difference between the horizontal columns at the 0.05 level.

*Total cost-state-0 (<17,548.3355). Total cost-state-1 (≥ 17,548.3355).

According to the combination of indicators, we got the following results: (1) for LOS group: “urine volume_length of operation _100-High,” “intraoperative ETCO2-0,” “crystalloid solution: colloid solution ratio −2:1-Less,” “intraoperative MBP-Normal,” “positive balance-1,” “whether to use opioids as dominance in postoperative analgesia (fentanyl)-N,” “intraoperative DBP-Normal,” “intraoperative SBP-Normal,” “postoperative compound nerve block analgesia-N,” P < 0.05 (see Table 5); (2) for TC group: “whether to use non-steroidal drugs in postoperative analgesia-Y,” “whether to use the dexmedetomidine-Y,” “intraoperative use of myocardial nutritional drugs-N,” “whether to use blood transfusion in 6–9 g hemoglobin-2,” “whether to use dexamethasone-N,” “crystalloid solution: colloid solution ratio −2:1-Low,” “intraoperative ETCO2-0,” “postoperative compound nerve block analgesia-Y,” “intraoperative SBP-Normal,” “intraoperative MBP-Normal,” “intraoperative use of hemostatic-N,” “intraoperative DBP-Normal,” P < 0.05 (see Table 5).

**TABLE 5:** The influence of the combination of multiple indicators for the postoperative length of stay and total cost.

Indicator		State 0	Proportion	State 1	Proportion	P
**Postoperative length of stay**						
Urine volume _ length of operation _100	High	642	67.08%	306	54.84%	0.000002
Intraoperative ETCO2	0	411	42.95%	171	30.65%	0.000002
Crystalloid solution: colloid solution ratio −2:1	Less	165	17.24%	78	13.98%	0.095058
Intraoperative mean blood pressure	Normal	147	15.36%	45	8.06%	0.000038
Positive balance	1	105	10.97%	39	6.99%	0.010791
Whether to use opioids as dominance in postoperative analgesia (fentanyl)	N	105	10.97%	36	6.45%	0.003489
Intraoperative diastolic blood pressure	Normal	84	8.78%	33	5.91%	0.044027
Intraoperative systolic blood pressure	Normal	63	6.58%	24	4.30%	0.065549
Postoperative compound nerve block analgesia	N	42	4.39%	12	2.15%	0.023422
Whether to use non-steroidal drugs in postoperative analgesia	N	21	2.19%	6	1.08%	0.1441
Intraoperative use of myocardial nutritional drugs	N	15	1.57%	3	0.54%	0.074374
Whether to use the dexmedetomidine	Y	9	0.94%	3	0.54%	0.3936
Intraoperative use of propofol	Y	6	0.63%	3	0.54%	0.827249
Whether to use blood transfusion in 6–9 g hemoglobin	2	3	0.31%	3	0.54%	0.502826
Whether to use dexamethasone	N	3	0.31%	3	0.54%	0.502826
Intraoperative use of sevoflurane	N	3	0.31%	0	0.00%	0.30156
Whether to use a muscle relaxant antagonist	Y	0	0.00%	0	0.00%	
Intraoperative use of hemostatic	N	0	0.00%	0	0.00%	
**Total cost**						
Whether to use non-steroidal drugs in postoperative analgesia	Y	561	66.79%	171	25.11%	7.47E-59
Whether to use the dexmedetomidine	Y	384	45.71%	132	19.38%	4.03E-27
Intraoperative use of myocardial nutritional drugs	N	381	45.36%	105	15.42%	1.37E-35
Whether to use blood transfusion in 6-9 g hemoglobin	2	378	45.00%	99	14.54%	3.87E-37
Whether to use dexamethasone	N	195	23.21%	57	8.37%	9.72E-15
Crystalloid solution: colloid solution ratio −2:1	Low	99	11.79%	6	0.88%	7.31E-17
Intraoperative ETCO2	0	69	8.21%	0	0.00%	1.93E-14
Postoperative compound nerve block analgesia	Y	30	3.57%	0	0.00%	6.32E-07
Intraoperative systolic blood pressure	Normal	18	2.14%	0	0.00%	1.22E-04
Intraoperative mean blood pressure	Normal	18	2.14%	0	0.00%	1.22E-04
Intraoperative use of hemostatic	N	9	1.07%	0	0.00%	0.017642
Intraoperative diastolic blood pressure	Normal	9	1.07%	0	0.00%	0.017642
Positive balance	1	6	0.71%	0	0.00%	0.072087
Whether to use a muscle relaxant antagonist	Y	6	0.71%	0	0.00%	0.072087
Whether to use opioids as dominance in postoperative analgesia (fentanyl)	N	6	0.71%	0	0.00%	0.072087
Urine volume _ length of operation _100	High	3	0.36%	0	0.00%	0.327101
Intraoperative use of sevoflurane	N	3	0.36%	0	0.00%	0.327101
Intraoperative use of propofol	Y	3	0.36%	0	0.00%	0.327101

### Anesthesia Bayesian decision intervention model results for length of stay and total cost

Based on the anesthesia Bayesian decision intervention model, we constructed the DAG decision structure for gynecological tumor diseases. We would manually make some adjustments according to the actual clinical significance. For LOS (length of stay), we got the following indicators: “positive balance,” “intraoperative ETCO2,” “whether to use opioids as dominance in postoperative analgesia (fentanyl),” “crystalloid solution: colloid solution ratio −2:1,” “urine volume _ length of operation _100” and “postoperative compound nerve block analgesia.” For TC (total cost), the results of the indicators are as follows: “intraoperative mean blood pressure,” “intraoperative systolic blood pressure,” “whether to use dexamethasone,” “whether to use non-steroidal drugs in postoperative analgesia,” “whether to use blood transfusion in 6–9 g hemoglobin,” “whether to use dexmedetomidine,” “postoperative compound nerve block analgesia,” “intraoperative use of myocardial nutritional drugs,” and “crystalloid solution: colloid solution ratio −2:1.”

**FIGURE 5:**
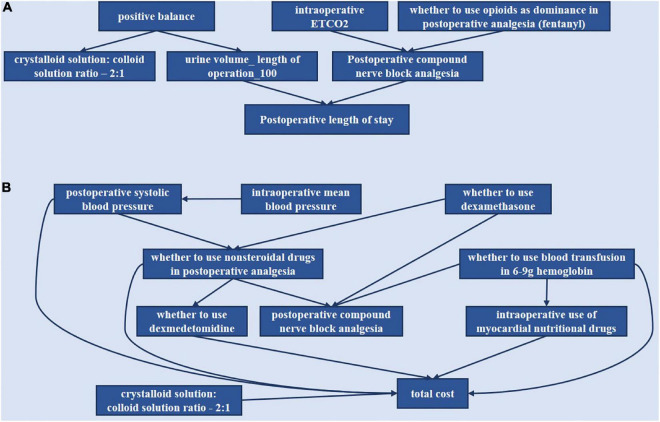
Bayesian causal network of various indicators of the postoperative length of stay and total cost. **(A)** Postoperative length of stay. **(B)** Total cost.

The relationships of the selected indicators are shown in Figure 5. As shown in Figure 5A, it included six indicators for the outcome, including postoperative length of stay. As shown in Figure 5B, the total cost included nine indicators for the outcome, total cost. The directed arc between nodes represented the dependency relationship among nodes.

“Positive balance” was the parent node of “crystalloid solution: colloid solution ratio −2:1” and “urine volume—length of operation −100.” “Intraoperative ETCO2” and “whether to use opioids as dominance in postoperative analgesia (fentanyl)” could affect LOS through modulation of “postoperative compound nerve block analgesia.”

“Whether to use dexamethasone” was the parent node of “whether to use non-steroidal drugs in postoperative analgesia” and “postoperative compound nerve block analgesia.” “Whether to use non-steroidal drugs” was the parent node of “whether to use dexmedetomidine,” “postoperative compound nerve block analgesia,” and “total cost.” “Intraoperative mean blood pressure” could affect “total cost” through “intraoperative systolic blood pressure,” which was the parent node of “whether to use non-steroidal drugs” and “total cost.” “Whether to use blood transfusion in 6–9 g hemoglobin” was the parent node of “intraoperative use of myocardial nutritional drugs,” “postoperative compound nerve block analgesia,” and “total cost.” Then “whether to use dexmedetomidine,” “intraoperative use of myocardial nutritional drugs,” and “crystalloid solution: colloid solution ratio −2:1” could directly affect “total cost.”

## Discussion

This study analyzed 1,827 patients undergoing laparoscopic gynecological surgery using BN based on 18 selected indicators related to anesthesia decision-making. Then, combining the selected indicators with specific grades and validation in patient data, we found that some of these impact indicators can improve the outcomes when combined. Finally, the DAGs of BN showed the interaction between the impact indicators through the arrows.

For the impact indicators we found, there is some clinical significance, and previous studies also proved it. For laparoscopic surgery, pneumoperitoneum is important and associated with ETCO2, and it did make a difference in our study. A previous study showed that low-pressure pneumoperitoneum was associated with improved parietal peritoneal perfusion (32), which may reduce clinically relevant complications. The “urine volume_length of operation_100” and “crystalloid solution: colloid solution ratio −2:1” also made sense. For urine volume, it can be influenced by fluid management such as restrictive fluid management strategy, open fluid management strategy or goal-directed fluid therapy (GDFT). Additionally, GDFT can reduce LOS and postoperative complications (33). At the same time, positive balance and blood pressure are also related to fluid management strategies. For an operative time, one study demonstrated that operative times > 120 min and 151–180 min, and ≥ 181 min predicted prolonged LOS (34). Another study indicated a significant association between high fluid volume given on the day of surgery and increased LOS and total costs (35). The above findings are consistent with our studies.

Differences also exist between our results and clinical practice. Our study recommended not using postoperative compound nerve block. However, nerve block has been reported to be effective in reducing pain and decreasing LOS or complication rates (36). Since the included patients are those with benign tumors undergoing laparoscopic surgery, they may not experience severe pain, and normal analgesic therapy can achieve satisfactory results. For TC, we found that indicators affecting total cost were mostly related to drug use. Our study recommends using dexmedetomidine to decrease TC. However, using common sense, we know that dexmedetomidine is expensive and leads to high costs. Perhaps, the cost could be decreased by reducing other complications. A previous study has shown that dexmedetomidine could attenuate the incidence of PONV (37).

Through the directed acyclic graph of BN analysis, we can predict the continuous impact of decisions on related indicators. Our study indicated that “positive balance” could affect the “crystalloid solution: colloid solution ratio −2:1” and “urine volume—length of operation −100” whist “urine volume—length of operation −100” can affect “postoperative length of stay.” It is consistent with clinical practice, while fluid management can influence patient recovery (35). “Intraoperative ETCO2” and “whether to use opioids as dominance in postoperative analgesia (fentanyl)” can affect “postoperative length of stay” by the modulation of “postoperative compound nerve block analgesia.” It has been proven that post-laparoscopic residual pneumoperitoneum volume was correlated with shoulder pain after laparoscopy (38, 39). Thus, “intraoperative ETCO2” was associated with analgesic planning. Then a study reported that multimodal analgesia improved postoperative pain control and minimized opioid-related adverse effects for all types of gynecological surgeries (14). This can also show that these indicators have interacted.

“Whether to use dexamethasone” was the parent node of “postoperative compound nerve block analgesia” and “whether to use non-steroidal drugs in postoperative analgesia,” the latter of which can affect “whether to use dexmedetomidine,” “postoperative compound nerve block analgesia” and “total cost.” This relationship suggests that the measures that control pain and postoperative nausea and vomiting affect the total cost. In addition, “intraoperative mean blood pressure” could affect “total cost” through “intraoperative systolic blood pressure,” which was the parent node of “whether to use non-steroidal drugs” and “total cost.” This correlation implies that blood pressure control may also influence the total cost. In addition, “crystalloid solution: colloid solution ratio −2:1” can independently affect “total cost.” So maybe there is room to explore the “crystalloid solution: colloid solution ratio −2:1” contribution to cost.

Machine learning has been applied to disease diagnosis, risk prediction, image recognition, and more. As a combination of probability theory and graph theory, BN has also played a corresponding role in the medical field (19, 40–44). However, the application of BN analysis for anesthesia decision-making in ERAS has not been reported yet. We investigated the indicators of anesthesia decision-making using BN analysis to identify the indicators that significantly impact anesthesia decision-making outcomes and study their combinations’ impact. Furthermore, the causal structure was explicitly represented, and the model can be learned from data, expert knowledge (no data), or a combination of the two approaches (19). Our research can be used to support clinical anesthesia decision-making based on the recommended indicators to achieve the optimal anesthetic part of ERAS.

However, our study still has some limitations. We only enrolled patients with benign tumors who participated in fewer ERAS measures. More patients diagnosed with other diseases must be included in the next step. In addition, the length of stay and total cost were analyzed separately, and the relationship between them was not explored. The next step is to investigate multiple outcomes in a single model. Moreover, this study was for anesthesiologists; we were most involved in intraoperative management and postoperative analgesia, which indicates a lack of preoperative measures. We need to include more indicators for the optimization of the whole ERAS.

## Conclusion

We used the BN to study the relationship between anesthesia-related ERAS measures, postoperative length of stay, and total cost. Some indicators can affect the outcomes, and their combinations can reduce LOS and TC. The relationship between the indicators in DAGs provides new ideas for clinical work. This strategy is a new attempt at the Bayesian algorithm in ERAS, breaking through traditional statistical methods’ limitations. It provides a feasible plan for the anesthesiologist to optimize ERAS protocols and make individualized decisions.

## Data availability statement

The data analyzed in this study is subject to the following licenses/restrictions: According to the regulation of the People’s Republic of China on the administration of human genetic resources, the collection of raw data was subject to approval by the science and technology administrative department of the state council (AQ: 2020SQCJ7444). Requests to access these datasets should be directed to BY, yibin1974@163.com.

## Ethics statement

The studies involving human participants were reviewed and approved by ethics committee of the First Affiliated Hospital of Army Medical University, ethics committee of Xuanwu Hospital of Capital Medical University, and ethics committee on Biomedical Research, West China Hospital of Sichuan University. Written informed consent for participation was not required for this study in accordance with the national legislation and the institutional requirements.

## Author contributions

BY, KW, and YC: study concept and design. YZ, YC, KZ, XS, DW, PD, XB, LZ, TZ, ZY, and YL: acquisition of data. YZ, YC, KZ, ZY, and YL: analysis and interpretation of data. JZ, YC, JG, and KL: technical support. BY and YC: funding acquisition. YZ, YC, and KZ: writing original manuscript. ZY and YL: revision of manuscript. All authors contributed to the article and approved the submitted version.
